# Recent trends in crystallography – a current IUCr journals perspective

**DOI:** 10.1107/S2052252519014507

**Published:** 2019-11-01

**Authors:** Andrew J. Allen

**Affiliations:** a Materials Measurement Science Division, National Institute of Standards and Technology, 100 Bureau Drive, Gaithersburg, MD 20899, USA

**Keywords:** crystallography, editorial

## Abstract

This Editorial considers the impact of recent work published in **IUCrJ** and other IUCr journals, as well as the relationship between **IUCrJ** and the other journals, in terms of where the most cited recent papers are used.

The IUCr publishes papers in nine scientific journals, including its high-impact, cross-cutting open-access journal, **IUCrJ**, plus its data publication, *IUCrData*. Where are these papers cited? To what degree are papers in **IUCrJ** cited in other IUCr journals, and *vice-versa*? What trends in crystallography can be discerned from either this or other information? In this Editorial, I attempt to provide some answers based on a brief survey of the most cited papers published since the beginning of 2018 in **IUCrJ**, as well as in the other IUCr journals. None of these papers yet contribute to IUCr journal performance metrics such as impact factor – but they will do so.

All six of the broad subject areas covered by **IUCrJ** are represented among its most highly cited recent papers. While I consider each area in turn, significant overlap in the subject areas covered by any one paper is both inevitable and desirable.

The most cited recent **IUCrJ** paper in the area of *Biology and medicine* is that by Guo *et al.* (2018 ▸) from the USA. Their paper, ‘Sample manipulation and data assembly for robust microcrystal synchrotron crystallography’, describes the development of micrometre-sized polyimide well mounts for manipulation of microcrystals, and their application in a robust data analysis for assembly of rotational microdiffraction data sets. It shows that microcrystals can be routinely utilized for acquisition and assembly of complete data sets from synchrotron microdiffraction beamlines. The paper has been cited in **IUCrJ**, *Acta Crystallographica D* and *Journal of Synchrotron Radiation*, as well as journals such as *Molecules* and *Communications Biology*.

In *Chemistry and crystal engineering*, the most cited recent paper is by Fugel *et al.* (2018 ▸) with authors from Germany, Denmark, Switzerland, the UK, Australia and Japan: ‘Probing the accuracy and precision of Hirshfeld atom refinement with *HARt* interfaced with *Olex*’. This paper focuses on Hirshfeld atom refinement, an X-ray structure refinement technique employing aspherical atomic scattering factors obtained from theoretically determined static electron densities. The method overcomes several limitations of independent atom modeling, and its accuracy and precision compare well with those obtained from neutron diffraction data or established multipole modeling of X-ray diffraction (XRD) data interpretations. As well as citations in *Acta Crystallographica A* and *B*, the paper has been cited in journals such as *CrystEngCom*, *Crystal Growth and Design*, *Crystallography Reviews* and *Journal of Physical Chemistry A*.

Cryogenic electron microscopy (*Cryo-EM*) was added to the **IUCrJ** remit in 2017 and has emerged as a new high-impact area for crystallography. Two 2019 *Cryo-EM* papers are among the most cited recent **IUCrJ** papers. In their paper: ‘Nanobeam precession-assisted 3D electron diffraction reveals a new polymorph of hen egg-white lysozyme’ Lanza *et al.* (2019 ▸), a group from Italy, present the first new protein structure both determined from 3D electron diffraction data and validated by X-ray crystallography. The authors point to potential new insights being achievable by combining electron and X-ray crystallography methods. Cited in **IUCrJ** and *Acta Crystallographica B* and *D*, this paper has also been cited in *ACS Central Science*, *Science Advances*, and in *Angewandte Chemie – International Edition*. The other paper highlighted here is from Zivanov *et al.* (2019 ▸) from a UK-based group: ‘A Bayesian approach to beam-induced motion correction in cryo-EM single-particle analysis’. The paper presents a new method to estimate particle motion trajectories and the amount of cumulative beam damage in cryo-EM single-particle analysis. The method is shown to outperform existing methods. Cited in **IUCrJ** and *Acta Crystallographica D*, its impact is also shown by citations in *Science*, *Nature* (and sister journals), *Journal of Structural Biology* and *Proceedings of the National Academy of Sciences* (*PNAS*).

*Materials and computation* encompasses a wide range of topics, and several recent papers are becoming well cited. Among these is the paper by Li *et al.* (2018 ▸), with authors from China, Mexico and France: ‘Phase transition and magneto­caloric properties of Mn_50_Ni_42 − *x*_Co_*x*_Sn_8_ (0 ≤ *x* ≤ 10) melt-spun ribbons’. This comprises in-depth studies of strong magnetostructural coupling in Mn–Ni–Co–Sn ribbons, showing them as potential candidates for magnetic refrigeration. Apart from **IUCrJ**, this paper has been cited in the materials and physics literature, including in *Scripta Materialia*, *Acta Materialia*, *Advances in Electronic Materials*, *Results in Physics*, *Journal of Alloys and Compounds*, *Applied Physics Letters*, *Journal of Magnetism and Magnetic Materials* and *Intermetallics*. Another paper to highlight here is that by Liu *et al.* (2018 ▸), a group from China: ‘Tailoring structural and magnetic properties of Mn_3 − *x*_Fe_*x*_Ga alloys towards multifunctional applications’. This also comprises in-depth studies of the structural and magnetic properties of Mn–Fe–Ga alloys, and shows how this group of alloys displays a wide range of multi-functionalities attributed to the tunable crystal structure. This paper has been cited in many of the same journals listed for Li *et al.* Two other recent papers should be mentioned briefly: Savitz *et al.* (2018 ▸), with authors from the USA and Israel: ‘Multiple-scale structures: from Faraday waves to soft-matter quasicrystals’, and Nakano *et al.* (2018 ▸) with authors from Japan: ‘Pressure-induced coherent sliding-layer transition in the excitonic insulator Ta_2_NiSe_5_’. Like many **IUCrJ** papers in this area, these papers are attracting citations from high-impact materials science and condensed matter physics journals.

The most cited recent **IUCrJ** paper in *Neutron and synchrotron science and technology* is by Tinti *et al.* (2018 ▸) with authors from Switzerland and Netherlands: ‘Electron crystallography with the EIGER^1^ detector’, which demonstrates the potential of a high-resolution 2D X-ray detector for rapid electron crystallography using electron energies up to 200 keV. Apart from citations in **IUCrJ**, *Acta Crystallographica A*, *B* and *D*, and *Journal of Applied Crystallography*, it has been cited in *ACS Central Science*, *Ultramicroscopy* and *Angewandte Chemie – International Edition*.

Finally, in the *Physics and free-electron laser (FEL) science and technology* area, two most cited recent papers should be highlighted. Halsted *et al.* (2018 ▸), with authors from the UK and Japan: ‘An unprecedented di­oxy­gen species revealed by serial femtosecond rotation crystallography in copper nitrite reductase’ presents high-impact results from FEL-based research. Using serial femtosecond rotation crystallography, an X-ray damage-free structure of the as-isolated copper nitrite reductase (CuNiR) was visualized. The work provides long-awaited clear-cut evidence for the mode of O_2_ binding in CuNiRs. Cited in **IUCrJ** and *Acta Crystallographica D*, the paper is also cited in *Philosophical Transactions of the Royal Society A*, *Scientific Reports*, *Analytical Bioanalytical Chemistry* and *ACS Catalysis*. The other most cited recent paper in this area is Wiedorn *et al.* (2018 ▸), with authors from Germany, the USA, Spain and Australia: ‘Rapid sample delivery for megahertz serial crystallography at X-ray FELs’. Results are presented of a liquid microjet, FEL-based megahertz serial diffraction experiment using soft X-rays, and the feasibility is outlined for FEL-based megahertz serial crystallography measurements with hard X-rays. This recent paper has attracted citations in *Acta Crystallographica D*, *Physical Review E*, *Physical Review Fluids*, *Applied Surface Science*, *Review of Scientific Instruments*, *International Journal of Molecular Sciences* and *Nature Communications*.

This brief survey of well cited, recent papers strongly shows how **IUCrJ** fulfills its goals of publishing open-access, high-quality and cross-cutting papers from all aspects of crystallography, and from all parts of the world active in crystallography. **IUCrJ** papers are cited in other IUCr journals, and the reverse is clearly true. Indeed, it is of interest to extend this survey to include well cited, recent papers in other IUCr journals in the context of the developing relationship between them and **IUCrJ**.

The three most cited recent papers (published since January 2018) in *Foundations and Advances (Acta Crystallographica A)* are: Miranda & Sasaki (2018 ▸) with authors from Brazil: ‘The limit of application of the Scherrer equation’; Hicks *et al.* (2018 ▸) with authors from the USA, Israel and Germany: ‘*AFLOW-SYM*: platform for the complete, automatic and self-consistent symmetry analysis of crystals’; and Steurer (2018 ▸), an open-access paper from Switzerland: ‘Quasicrystals: What do we know? What do we want to know? What can we know?’. Together, these papers are cited in a wide range of journals, including *CrystEngComm*, *Journal of Nanoparticle Research*, *MRS Bulletin*, *Acta Materialia*, *Nature Communications* and *Physical Review Letters*, as well as *Journal of Applied Crystallography* and *Acta Crystallographica A*.

The two most cited recent papers in *Structural Science, Crystal Engineering and Materials (Acta Crystallographica B)* are: Gagné & Hawthorne (2018 ▸), an open-access paper with authors from Canada: ‘Bond-length distributions for ions bonded to oxygen: metalloids and post-transition metals’; and Goel *et al.* (2018 ▸) with authors from India and Portugal: ‘X-ray, dielectric, piezoelectric and optical analyses of a new nonlinear optical 8-hy­droxy­quinolinium hydrogen squarate crystal’. Apart from citations in *Acta Crystallographica B*, *C* and *E*, the former has been cited in journals such as *Journal of Solid-State Chemistry* and *Chemistry in Materials*, the latter in *Journal of Alloys and Compounds*, *Chemical Physics Letters* and *Ionics*.

The three most cited recent papers in *Structural Chemistry (Acta Crystallographica C)* are: Zhao *et al.* (2018 ▸), from China: ‘Novel tantalum phosphate Na_13_Sr_2_Ta_2_(PO_4_)_9_: synthesis, crystal structure, DFT calculations and Dy^3+^-activated fluorescence performance’; Minyaev *et al.* (2018 ▸) from Russia: ‘Isomorphous rare-earth tris­[bis­(2,6-diiso­propyl-phenyl) phosphate] complexes and their catalytic properties in 1,3-diene polymerization and in the inhibited oxidation of polydi­methyl­siloxane’; and Yao *et al.* (2018 ▸) also from China: ‘Novel asymmetric 3,5-bis­(aryl­idene)piperidin-4-one derivatives: synthesis, crystal structures and cytotoxicity’. These in-depth articles have been cited in *Acta Crystallographica C*, itself, and elsewhere in journals such as *Journal of Alloys and Compounds*, *Inorganic Chemistry* and, for Yao *et al.*, *European Journal of Medicinal Chemistry*.

The two most cited recent papers in *Structural Biology (Acta Crystallographica D)*, both open access, are Winter *et al.* (2018 ▸) with authors from the USA and the UK: ‘*DIALS*: implementation and evaluation of a new integration package’; and Afonine *et al.* (2018 ▸) with authors from the USA, China, the UK and France: ‘Real-space refinement in *PHENIX* for cryo-EM and crystallography’. These two papers, both centered on software development, comprise two of the three most cited recent papers published in any IUCr journal. Both papers have been cited in **IUCrJ**, *Acta Crystallographica A*, *D* and *F*, *Journal of Applied Crystallography*, and *Journal of Synchrotron Radiation*, as well as in *CrystEngCom*, countless biology, biochemistry and medical research journals, as well as high-impact publications in *Nature* and *Science*.

Among other things, *Crystallographic Communications (Acta Crystallographica E)* provides a rapid and economical open-access publication route for authors from developing countries. The most cited recent paper is Tan *et al.* (2019 ▸) with authors from Malaysia and India: ‘Utilizing Hirshfeld surface calculations, non-covalent interaction (NCI) plots and the calculation of interaction energies in the analysis of molecular packing’. This has been cited in *Acta Crystallographica C* and *E*, as well as in *Zeitschrift für Kristallographie-Crystalline Materials*.

The most cited recent paper in *Structural Biology Communications (Acta Crystallographica F)* is a brief Topical Review by Blaum *et al.* (2018 ▸) with authors from Germany: ‘Spin ballet for sweet encounters: saturation-transfer difference NMR and X-ray crystallography complement each other in the elucidation of protein–glycan interactions’. It has been cited in *Acta Crystallographica F*, and other journals including *Angewandte Chemie – International Edition*, *Journal of Physical Chemistry Letters* and *ChemComm*.

The three most cited recent papers in the *Journal of Applied Crystallography* are Coehlo (2018 ▸) from Australia: ‘*TOPAS* and *TOPAS*-*Academic*: an optimization program integrating computer algebra and crystallographic objects written in C++’ (most cited of all the recent papers in IUCr journals and cited in all IUCr journals except *Acta Crystallographica D*, *E* and *F*); Wood *et al.* (2018 ▸) with authors from Australia and Taiwan: ‘QUOKKA, the pinhole small-angle neutron scattering instrument at the OPAL Research Reactor, Australia: design, performance, operation and scientific highlights’ (part of the recent Neutron Scattering Special Issue); and Narayanan *et al.* (2018 ▸), an open-access paper from France: ‘A multipurpose instrument for time-resolved ultra-small-angle and coherent X-ray scattering’. The Coelho paper has been cited in many high-impact journals such as *Advanced Materials*, *Angewandte Chemie – International Edition*, *Chemistry in Materials*, *Journal of the American Chemical Society*, *Nature Materials*, *Physical Review Letters*, *Langmuir*, and a broad range of journals in the physical, chemical, biological and material sciences. Together, the other two papers on neutron and X-ray beamlines, respectively, have been cited in *Journal of Applied Crystallography*, *Journal of Synchrotron Radiation* and *Acta Crystallographica A*, as well as in the chemical and soft matter literature, including *Journal of Colloid and Interface Science*, *Biomacromolecules*, *ACS Sustainable Chemistry and Engineering*, *Particles and Particle Systems Characterization* and *ACS Nano*.

The three most cited recent papers (all open access) in *Journal of Synchrotron Radiation* are Owada *et al.* (2018 ▸) from Japan: ‘A soft X-ray free-electron laser beamline at SACLA: the light source, photon beamline and experimental station’; Wojdyla *et al.* (2018 ▸) from Switzerland: ‘*DA+* data acquisition and analysis software at the Swiss Light Source macromolecular crystallography beamlines’; and Sala *et al.* (2018 ▸) with authors from France, Spain Lebanon, Finland and Italy: ‘A high-energy-resolution resonant inelastic X-ray scattering spectrometer at ID20 of the European Synchrotron Radiation Facility’. Together, these papers are cited in **IUCrJ**, *Acta Crystallographica B* and *D*, *Journal of Applied Crystallography*, and the *Journal of Synchrotron Radiation*. Elsewhere, these papers have been cited across a wide range of physics, materials, chemistry and biology research journals, including *Physical Review Letters*, *Physical Review A* and *B*, *Physical Review Accelerators and Beams*, *Applied Physics Letters*, *Nature Communications*, *Nature Methods*, *Science Advances*, *Cell*, and *eLife*.

IUCr’s fully open-access data publication, *IUCrData*, provides short descriptions of crystallographic datasets, and facilitates access to the data. While originally associated with *Acta Crystallographica E*, various data paradigms that could connect *IUCrData* to other IUCr journals are being considered. Two cited recent data publications, both from India, are Aarthi *et al.* (2018 ▸): ‘(4-Methyl­phenyl)­methanaminium bromide hemihydrate’ and Muthuselvi *et al.* (2018 ▸): ‘60-(3-Bromo­phenyl)-70-nitro-10,60,70,7a′-tetra­hydro-30*H*-spiro[indeno­[1,2-*b*]quinoxaline 11,50-pyrrolo-[1,2-*c*]thia­zole]’.

Measured by use of well cited **IUCrJ** papers in other IUCr journals, and *vice versa*, together with the overall citation reach of high-quality papers published in all IUCr journals, I conclude the health of IUCr journal publication, including how **IUCrJ** relates to the other journals, to be excellent. Regarding future developments, I draw attention to the appointment of three new Commissioning Editors in *Biology and Biological Sciences*, *Chemical Crystallography* and *Materials, Methods and Instrumentation*. Two have been appointed: Professor Robert Steiner (King’s College, London, UK) for *Biology and Biological Sciences*, and Professor Elena Boldyreva (Novosibirsk State University, Novosibirsk, Russia) for *Chemical Crystallography*. We hope to appoint a Commissioning Editor for *Materials, Methods and Instrumentation*, shortly. These Commissioning Editors will work across all IUCr journals commissioning high-quality articles and Special Issues. This will strengthen relationships among the journals regarding scope and content development.

Finally, I draw attention to the upcoming IUCr 2020 Congress to be held in August 2020 in Prague, Czech Republic. As a member of the International Program Committee (IPC), I was privileged to attend the IPC planning meeting in Prague in May 2019. A Congress program is being assembled that promises to be stunningly rich and diverse in subject distribution, and in gender and geographical origin of the speakers. Plenary and Keynote Speakers and (when selected) the microsymposia speakers will be invited to submit research or topical review papers associated with their presentations. We look forward to receiving their high-quality submissions both to **IUCrJ** and to other IUCr journals.

## Footnotes

1 Any mention of commercial products is for information only; it does not imply recommendation or endorsement by NIST.
